# A Remedy for the Painful Affection Produced from Cutting the Lower Dens Sapientia or Wisdom Tooth, &c.

**Published:** 1843-09

**Authors:** J. S. Gunnel


### ARTICLE IV.

*A remedy for the painful affection produced from cutting the lower dens sapientia or wisdom tooth, &c.* Read before the American Society of Dental Surgeons, at its fourth Annual Meeting; Baltimore, July 20th, 1843. By J. S. GUNNEL, M. D.

HAVING seen several cases of severe inflammation of the gums and the other soft parts surrounding the under wisdom teeth or dens sapientia, at the time of cutting those teeth; soon after I commenced the practice of dentistry (about 1820, and having also been a great sufferer in that way myself previously to my studying medicine,) I was led to seek a remedy for that painful affection; this inflammation is sometimes so great as to produce extreme pain at the junction of the jaws, and so much swelling and soreness of the surrounding soft parts (and extending to the throat) that the patient is hardly able to open the mouth, so as to give the physician an opportunity of even seeing the parts affected.

In such extreme cases I have used the depleting remedies both general and local with good effect; but my object at present is to show how to prevent such cases or their recurrence, by pointing out their causes and applying the remedy in time.

The cause of this painful affection when in its extreme cases, arises generally from the upper wisdom tooth having cut through the gum first (before the under wisdom tooth of the same side) and passing so low down as to rest on the lower gum, before the under wisdom tooth begins to cut through the gum; the upper wisdom tooth in some cases being from an eighth to a fourth of an inch lower (or longer) at its cutting (or grinding) point than the molares that is next to it; now it will be readily perceived, that the first move of the lower wisdom tooth to cut through the gum (or any food or other substance pressing thereon or the cheek or soft parts folding over or between the teeth) will produce irritation and inflammation which is aggravated by pressing the jaws together, and when the soft surrounding parts are once swelled they will frequently press forward over the jaws or tooth, so as to be pressed on when the mouth is closed, thus continually irritating the inflamed parts.

In some cases I have cut off the whole of the gum covering the top of the under tooth, with great advantage; but occasionally the tooth is so very far back, that the flesh back of the tooth and at the side, will press over the under tooth, and still continue to be pressed upon or mashed, when the jaw closes, so as to produce great inflammation of the surrounding parts, extending to the throat, and in some cases accompanied with great stiffness of the joint of the jaw. In all such cases as above, the upper wisdom tooth should be extracted as soon as possible, and the cure will soon be completed and the recurrence ever after prevented, and the under tooth be freed from difficulty in arriving at its proper situation.

The above plan of cure I would recommend with great confidence, as I have seen a number of persons that had been occasionally sufferers for years, from the above affection, though they had been attended by some of the best physicians and dentists in this country, and some in Europe, one gentleman an European told me that his case had cost two hundred dollars in physician's fees, and a loss of business of at least a thousand dollars. This case as well as others was readily cured by extracting the upper wisdom tooth, and the use of some mild astringent and mucilaginous wash for the inflamed parts. In those cases the upper wisdom teeth are located so very far back as not to be considered a loss from extraction.

